# Your friendly neighborhood spider bite – Or not: A fishy case of *Mycobacterium marinum*

**DOI:** 10.1016/j.idcr.2026.e02671

**Published:** 2026-07-08

**Authors:** Briccio Cadiz, Katherine Raymer, Zackary Houghton, Riley Bricker, Marlon C. Torrento, Nickolas A. Bacon, Vikram Oke, Karthik V. Iyer

**Affiliations:** a Department of Internal Medicine, Mercy Hospital Jefferson, Festus, MO, USA; b Lake Erie College of Osteopathic Medicine, Seton Hill, Greensburg, PA, USA; c Lake Erie College of Osteopathic Medicine, Erie, PA, USA; d Department of Infectious Disease, Mercy Hospital Jefferson, Festus, MO, USA; e Southern Missouri Infectious Disease Specialists, Festus, MO, USA

**Keywords:** *Mycobacterium marinum*, Nontuberculosis mycobacterium, Cutaneous infection, Diagnostic anchoring, Aquarium granuloma, Immunocompromised host, Outpatient parenteral antimicrobial therapy

## Abstract

*Mycobacterium marinum* is a slow-growing nontuberculous mycobacterium, most commonly causing cutaneous infection after prolonged or repeated exposure to contaminated aquatic environments. Diagnosis is frequently delayed because of its indolent course, nonspecific clinical features, and frequent misattribution to more common dermatologic conditions. We report the case of a 51-year-old immunocompromised woman who developed a progressive ulcerative lesion on the right wrist after a presumed spider bite following a farm visit. Despite multiple emergency department encounters and empiric antibacterial therapy, the lesion failed to improve. Acid-fast bacilli culture from a wound specimen ultimately grew *M. marinum* several weeks after initial presentation. Subsequent history revealed a previously undisclosed history of exposure to a home aquarium. Management was complicated by impaired oral antimicrobial absorption following prior bariatric surgery and incomplete clinical response to initial therapy, necessitating prolonged outpatient parenteral antimicrobial treatment, with eventual clinical resolution. This case underscores the impact of diagnostic anchoring bias, highlights the importance of thorough environmental exposure assessment beyond classic aquatic settings, and emphasizes early consideration of nontuberculous mycobacterial infection in chronic, nonhealing cutaneous lesions.

**Key Message**

Cutaneous *Mycobacterium marinum* infection can masquerade as spider envenomation. Inland home aquarium exposure, immunosuppression, and post-bariatric oral malabsorption may each complicate diagnosis and treatment. Early AFB cultures and host-tailored therapy are essential to avoid prolonged morbidity.

**Teaching Points**

• Chronic, nonhealing cutaneous lesions that fail empiric antibacterial therapy should prompt early consideration of nontuberculous mycobacterial infection.
• Absence of classic aquatic exposure does not exclude *Mycobacterium marinum*; detailed environmental history may reveal delayed or overlooked risk factors.
• Normal inflammatory markers do not rule out indolent atypical infections.
• Host factors, including immunosuppression and impaired oral absorption, may necessitate deviation from standard oral treatment regimens.

## Introduction

*Mycobacterium marinum* is a slow-growing nontuberculous mycobacterium (NTM) found in fresh, salt, and brackish water environments and is a leading cause of extrapulmonary NTM infection worldwide [1], [2]. Human infection typically follows minor skin trauma with subsequent inoculation from contaminated water or aquatic animals, most commonly affecting the upper extremities [1], [2], [3], [4].

Despite these characteristic associations, diagnosis is frequently delayed. Clinical presentation is often indolent and nonspecific, inflammatory markers are commonly normal, and lesions are frequently misattributed to bacterial cellulitis, inflammatory dermatoses, or arthropod bites [3], [4], [5]. Definitive diagnosis relies on mycobacterial culture, which requires prolonged incubation at lower temperatures (typically between 30 and 32°C), further contributing to diagnostic delay [1], [2].

Although reported incidence remains low, *M. marinum* infection is likely underdiagnosed, particularly in immunocompromised individuals and inland populations without recognized aquatic occupational exposure, where clinician awareness and targeted microbiologic testing are less consistent. We present a case that is distinct from prior reports in three respects: the exposure source was a home aquarium not initially disclosed, the patient was deeply immunocompromised with multiple comorbidities, and prior bariatric surgery necessitated intravenous rather than standard oral antimycobacterial therapy. This constellation of factors illustrates how geographic, historical, and host-related assumptions can compound diagnostic delay and treatment failure in cutaneous *M. marinum* infection.

### Case Presentation

**Fig. 1:**
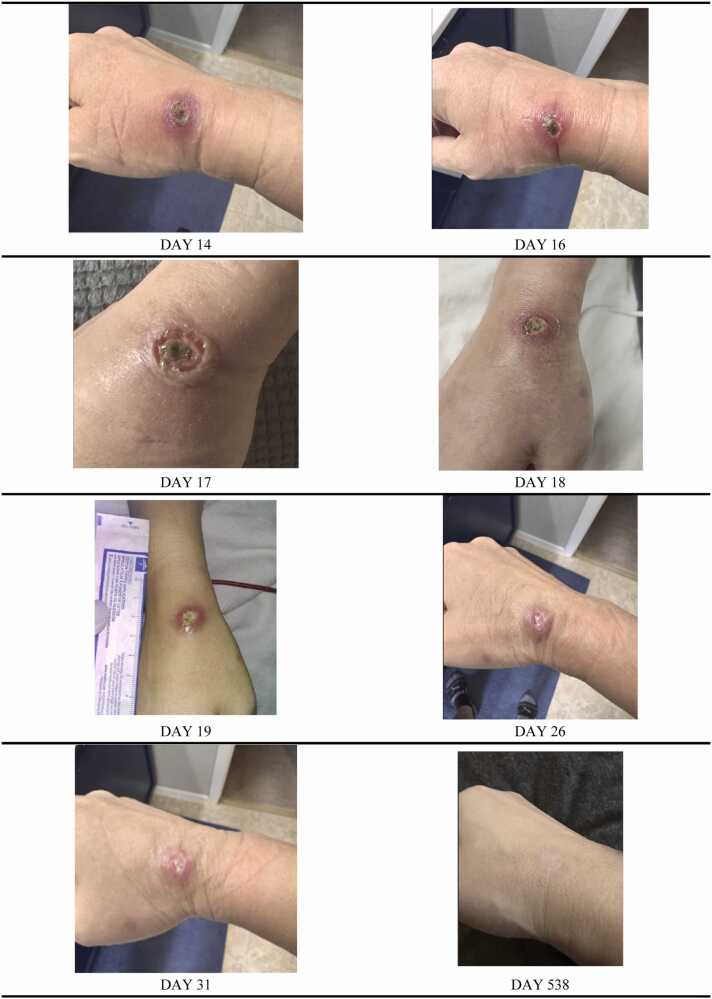
Serial image progression of cutaneous *Mycobacterium marinum* infection. Representative images demonstrating evolution of the right wrist lesion following symptom onset (Day 0). No image was available from Day 0, when a small pustule initially appeared. Panels depict progression from early ulceration with surrounding erythema (Days 14–19) through partial improvement (Days 26 and 31) and complete resolution with residual scarring approximately 18 months after symptom onset (Day 538).

A 51-year-old woman with a medical history of systemic lupus erythematosus, mixed connective tissue disease, Sjögren syndrome, hypothyroidism, asthma, prior estrogen receptor–positive breast cancer, and multiple abdominal surgeries including gastric sleeve and gastric bypass presented with a progressively worsening lesion on the right lateral wrist (Fig. 1). She was receiving immunosuppressive therapy.

Three days after visiting a farm in the Midwestern United States, she noted a tender bump on the wrist, attributed to an unwitnessed spider bite. Over several days, the lesion enlarged and became increasingly painful. She attempted self-incision and drainage using a lancet with local cleansing, after which the lesion worsened. Self-incision of the lesion with a lancet prior to microbiologic evaluation likely disrupted the dermal barrier and facilitated inoculation of organisms into deeper soft tissue planes, potentially accelerating progression from superficial pustule to ulcerative disease with sinus tract formation.

During the subsequent two weeks, she presented to the emergency department on multiple occasions. Initial evaluations resulted in normal lab values (Table 1) and empiric treatment was initiated with doxycycline, topical mupirocin, and oral cephalexin without improvement. The lesion progressed to ulceration with surrounding erythema and proximal streaking. Despite the absence of witnessed arthropod exposure, the lesion continued to be attributed to presumed spider envenomation. During a subsequent visit, she received intravenous vancomycin but was discharged, with continued progression.

**Table 1:** Summary table displaying the lab values obtained during multiple examinations, including emergency department (ED) visits and hospital admissions. Day 0 represents the day of symptom onset.

	Day 7 (ED Visit 1)	Day 12 (ED Visit 2)	Day 18 (Hospital Admission)	Day 19	Day 20	Day 21	Day 22 (Discharged from Hospital)	Reference Ranges
**Metabolic/Electrolytes**								
Sodium	136	137	138	142	138	141	-	136–145 mmol/L
Potassium	4.0	4.4	4.7	4.0	3.7	4.1	-	3.4–5.1 mmol/L
Chloride	100	101	104	105	105	105	-	98–107 mmol/L
CO2	26	23	27	26	23	26	-	22–29 mmol/L
BUN	21	17	24	19	14	14	-	6–20 mg/dL
Creatinine	0.78	0.75	0.84	1.00	0.70	0.76	-	0.51–0.95 mg/dL
Total Bilirubin	< 0.2	0.2	0.2	-	-	0.2	-	0.3–1.2 mg/dL
ALP	66	70	74	-	-	58	-	40–150 U/L
AST	40	36	46	-	-	37	-	0–33 U/L
ALT	24	30	28	-	-	32	-	0–33 U/L
**Hematology**								
Hemoglobin	12.2	12.6	13.0	12.5	12.3	12.2	12.9	11.9–15.1 g/dL
Hematocrit	37.0	39.5	40.7	39.2	38.0	38.2	40.3	38–47%
WBC	5.7	5.5	6.2	5.6	6.6	7.1	7.1	4.5–10.5 K/uL
Platelets	244	241	274	253	234	236	270	150–400 K/uL
**Inflammatory Markers**								
ESR	15	-	19	-	-	-	-	0–30 mm/Hr
CRP	< 3.0	-	< 3/0	-	-	-	-	< 8.0 mg/L
**Cultures**								
AFB Culture	-	-	-	Positive	-	-	-	Negative

**Abbreviations:** Hb = hemoglobin; Hct = hematocrit; WBC = white blood cell count; CRP = C-reactive protein; ALP = alkaline phosphatase; AST = aspartate transaminase; ALT = alanine transaminase; GFR = glomerular filtration rate; BUN = blood urea nitrogen; Na⁺ = sodium; K⁺ = potassium; Cl⁻ = chloride; CO₂ = bicarbonate; Glu = glucose; CK-MB = creatine kinase MB isoenzyme; aPTT = activated partial thromboplastin time; PT/INR = prothrombin time/international normalized ratio.

Eighteen days after symptom onset, her primary care physician evaluated her for a nonhealing ulcer and low-grade fevers and referred her for hospital admission due to failure of outpatient therapy and concern for impaired oral absorption. On admission, she was afebrile and denied systemic symptoms. Physical examination revealed an approximately 2 × 2 cm ulcer with surrounding erythema and a pale central base. Leukocyte count and inflammatory markers were within normal limits (Table 1).

Contrast-enhanced computed tomography demonstrated cellulitis with a small sinus tract extending into deeper soft tissues without abscess or osseous involvement. Magnetic resonance imaging of the right wrist subsequently demonstrated no involvement of the muscle, fascia, tendons, or bone, supporting a clinical impression of infection confined to the skin and superficial soft tissue. Wound cultures, including acid-fast bacilli cultures, were obtained. She was treated with intravenous vancomycin and piperacillin-tazobactam, later narrowed to cefazolin, along with wound care. She received two doses of intravenous methylprednisolone and was discharged on hospital day five with oral doxycycline and cephalexin.

Approximately four weeks after specimen collection, acid-fast bacilli culture returned positive for *Mycobacterium marinum* [1], [2]. Serial clinical photographs demonstrated progressive ulceration with surrounding erythema over the first three weeks following symptom onset (Fig. 1). Subsequent interviews revealed a history of home aquarium exposure. Patient was continued on oral doxycycline alone for the next 6 weeks. Due to incomplete clinical resolution and impaired oral absorption, she was initiated on outpatient parenteral antimicrobial therapy with intravenous doxycycline for an additional six weeks, resulting in gradual and complete resolution.

## Discussion

### Diagnostic anchoring and delay

This case demonstrates a common diagnostic pitfall in cutaneous *M. marinum* infection: anchoring bias toward a presumed spider bite despite lack of confirmatory exposure. Similar misattribution has been reported previously and contributes substantially to diagnostic delay [5], [6]. Routine laboratory studies are frequently normal, and standard bacterial cultures are unrevealing, further reinforcing premature diagnostic closure [1], [2], [3]. In this case, diagnostic anchoring persisted despite multiple emergency department encounters and an eventual hospital admission spanning more than two weeks, illustrating that repeated clinical contact does not necessarily prompt diagnostic reassessment once an initial impression has been established. Emerging data suggest that PCR-based species identification may reduce diagnostic delay and improve treatment accuracy compared with culture alone [7]. .

### Brown recluse spider bite mimicry

Cutaneous lesions caused by *Mycobacterium marinum* may closely resemble necrotic arachnid envenomation, particularly brown recluse (*Loxosceles reclusa*) bites, which are endemic to the Midwestern United States. These bites classically progress over 24–72 h from a mildly painful papule to a lesion with central pallor and surrounding erythema (the “red, white, and blue” sign), occasionally evolving into a necrotic ulcer with eschar; systemic symptoms such as low-grade fever are uncommon.

**Fig. 2:**
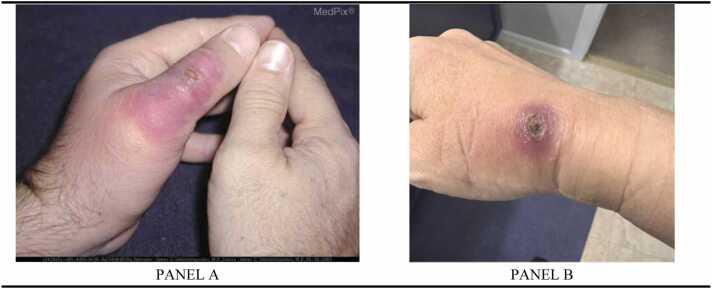
Clinical comparison of brown recluse envenomation and cutaneous *Mycobacterium marinum* infection. Panel A: Representative ulcerated lesion following brown recluse (*Loxosceles reclusa*) envenomation demonstrating central necrosis with surrounding erythema and induration, characteristic of cutaneous loxoscelism. Panel B: Patient lesion (Day 14) demonstrating a necrotic center with surrounding inflammatory border due to *Mycobacterium marinum* infection. Image source: MedPix® Medical Image Database, National Library of Medicine. Case ID: MPX2157. Used for educational purposes.

The lesion in our patient demonstrated a central ulcer with surrounding erythema and progressive necrosis, features that significantly overlap with reported brown recluse envenomation. In the absence of witnessed spider exposure, however, brown recluse bites are frequently overdiagnosed, and multiple infectious etiologies, including nontuberculous mycobacteria, should be considered in nonhealing or atypical lesions. This clinical overlap likely contributed to diagnostic anchoring and delayed microbiologic evaluation in this case (Fig. 2).

#### Literature review and clinical context

In a nationwide retrospective cohort study from Denmark, Holden et al. reported a median diagnostic delay of 194 days for culture-confirmed *M. marinum* infections, frequently misdiagnosed as bacterial cellulitis or inflammatory dermatoses and predominantly involving the upper extremities, with aquarium exposure as the most commonly identified source [5], [8]. .

Bridwell et al. described a case of *M. marinum* cellulitis initially misdiagnosed as a spider bite, leading to delayed AFB testing and prolonged empiric antibacterial therapy [6]. The present case extends these findings to a Midwestern setting with delayed recognition of aquatic exposure and host-specific treatment barriers, consistent with the heterogeneous regimens reported in an Italian multicenter series [8]. .

#### Host factors and treatment implications

Immunosuppression and impaired oral absorption following bariatric surgery likely contributed to treatment failure with oral therapy and necessitated prolonged outpatient parenteral antimicrobial therapy. While cutaneous *M. marinum* infection is often managed with oral agents, host factors may necessitate deviation from standard regimens [1], [2], [9], [10]. In the retrospective cohort study by Holden et al., broad spectrum antibiotics including doxycycline and cephalexin were started for broad spectrum coverage that included *Streptococcus pyogenes* and MRSA [5]. Although empiric broad-spectrum coverage is reasonable in this clinical context, such regimens are insufficient to eradicate *M. marinum*, which requires targeted antimycobacterial therapy of adequate duration. Bridwell et al. also noted that for deeper invasion of *M. marinum*, extended treatment with rifampin plus ethambutol with possible addition of clarithromycin is usually needed for adequate clearance [6]. .

Doxycycline was continued as monotherapy throughout the oral and intravenous treatment phases. Magnetic resonance imaging of the right wrist showed no involvement of the muscle, fascia, tendons, or bone, consistent with infection confined to the skin and soft tissue, for which monotherapy with a macrolide, tetracycline, or trimethoprim-sulfamethoxazole is a reasonable approach. Doxycycline was favored for its tolerability: macrolide therapy was avoided given a potential interaction between azithromycin or clarithromycin and the patient’s hydroxychloroquine, used for systemic lupus erythematosus, with attendant risk of QT prolongation, and trimethoprim-sulfamethoxazole was avoided after a prior emergency department visit for severe hypoglycemia attributed to its interaction with hydroxychloroquine. Because *M. marinum* is generally susceptible to standard first-line agents including macrolides and tetracyclines, susceptibility testing – typically reserved for treatment failure or relapse – was not pursued.

The two doses of intravenous methylprednisolone she received during the index hospitalization, prior to AFB culture positivity, were recommended by the wound care service for a then-presumed brown recluse spider envenomation. Administration of corticosteroids in the setting of an unrecognized mycobacterial infection warrants caution, as immunosuppression could theoretically impair containment of mycobacterial proliferation; in this case, however, the brief, limited-dose course did not appear to precipitate clinical worsening, and complete resolution was ultimately achieved with targeted antimycobacterial therapy.

Her slow initial response to oral doxycycline was attributed to impaired absorption related to her prior Roux-en-Y gastric bypass, prompting transition to intravenous therapy rather than substitution of an alternative oral agent. Malabsorptive bariatric procedures such as Roux-en-Y gastric bypass have been shown to alter the pharmacokinetics of several classes of oral antibiotics, including reduced bioavailability of certain beta-lactams and macrolides, supporting impaired enteral absorption as a plausible contributor to her incomplete response, although tetracyclines have not been as extensively studied in this population [13]. No doxycycline serum levels were obtained, so this mechanism remains inferred rather than confirmed.

Treatment recommendations for *M. marinum* remain heterogeneous (Table 2). In the nationwide Danish cohort study by Holden et al., treatment regimens varied considerably, with doxycycline or tetracyclines used in 48.1% of cases (87.5% resolution), rifampin–ethambutol combinations in 34.6% (63.6% resolution), and triple therapy reserved for more extensive disease [5]. These findings underscore the absence of a universally accepted first-line regimen and reflect clinician-driven tailoring based on severity and host factors.

**Table 2:** Summary of antimicrobial regimens and reported outcomes for cutaneous *Mycobacterium marinum* infection derived from published cohort and case series data. Data regarding antibiotic regimen frequency and clinical resolution rates are adapted from a nationwide Danish retrospective cohort study of culture-confirmed *M. marinum* infections (Holden et al.) [5]. Photodynamic therapy (PDT) outcomes are derived from a recent JAMA Dermatology case series evaluating PDT as monotherapy and adjunctive therapy (Hu et al.) [12]. Reported percentages reflect treatment utilization and documented clinical resolution within each study. These data illustrate heterogeneity in therapeutic approaches and support individualized, severity-based management strategies.

Severity/Setting	Drug Regimen (PO)	**Notes**
Mild/Localized	Doxycycline or Minocycline	First line for uncomplicated lesions
	Clarithromycin or TMP-SMX	Alternative or monotherapy
Moderate/Deep/Refractory	Rifampin + Clarithromycin	Combination therapy for deeper infection
	Rifampin + Ethambutol	Alternative Combination therapy
Severe/Extensive/Chronic	Rifampin + Ethambutol + Macrolide	Triple therapy if severe or slow response
Other Treatments noted in literature	Surgical Debridement	Abscess/Tenosynovitis/ or medical therapy failure
	Photodynamic Therapy (PDT) in conjunction with antibiotics	% Resolution of Ulcer
	• PDT Monotherapy (1–6 treatments)	94.1%
	• PDT + Doxycycline or Tetracycline	87.5%
	• PDT + Clarithromycin or Azithromycin	100%
	• PDT + Rifampicin and Ethambutol	63.6%
	• PDT + Clarithromycin and Ethambutol	60.0%
	• PDT + Rifampicin, Clarithromycin, and Ethambutol	55.6%

Although the Infectious Diseases Society of America (IDSA) has published comprehensive guidelines for several nontuberculous mycobacterial infections, there are currently no updated, dedicated IDSA guidelines specific to *Mycobacterium marinum.* Existing recommendations are derived from the 2007 American Thoracic Society/IDSA statement on NTM disease, which provides general guidance for cutaneous infection but does not establish standardized duration or regimen hierarchy [11]. This lack of definitive guidance contributes to variability in clinical practice and reinforces the importance of individualized treatment decisions.

Emerging adjunctive modalities have also been described. In a recent JAMA Dermatology study, Hu et al. reported successful use of photodynamic therapy (PDT) for localized cutaneous *M. marinum* infection, achieving 94.1% clearance (16 of 17 patients) when used as monotherapy [12]. PDT also reduced total antibiotic duration when combined with systemic therapy. These findings suggest that PDT may represent a viable antibiotic-sparing option in select immunocompetent patients with superficial disease, though larger studies are needed before routine adoption.

### Strengths and limitations

A notable strength of this report is the longitudinal photographic documentation of lesion progression over 538 days, which provides an unusually detailed visual record of the natural history and treatment response of cutaneous *M. marinum* in an immunocompromised host. Limitations include reliance on retrospective history-taking for exposure identification: the home aquarium was not disclosed until after the AFB culture had already returned positive, meaning this exposure history played no role in prompting the diagnostic workup and instead served only retrospectively to explain it, underscoring how heavily diagnosis in this case depended on culture-based testing rather than environmental history-taking alone. As discussed above, the absence of confirmatory pharmacokinetic data and the lack of formally standardized treatment guidance for *M. marinum*. Whether the 12-week treatment course (6 weeks oral therapy followed by 6 weeks of IV therapy via OPAT) represents the optimal duration cannot be determined from a single case.

### Lessons for clinical practice

Chronic, nonhealing cutaneous lesions that fail empiric antibacterial therapy should prompt early consideration of nontuberculous mycobacteria and acid-fast bacilli cultures. Absence of classic aquatic exposure should not exclude *M. marinum* from the differential diagnosis, particularly in immunocompromised patients [1], [2], [3], [5]. Occupational clusters and atypical exposure histories have been well documented, underscoring the importance of detailed environmental history taking [4], [5]. .

## Conclusion

Cutaneous *M. marinum* infection remains an important diagnostic consideration in chronic skin lesions with indolent progression. Diagnostic anchoring and delayed microbiologic testing can prolong morbidity and complicate management. Early clinical suspicion and timely acid-fast culture acquisition may improve outcomes. Clinicians practicing in non-coastal and inland settings should maintain awareness of *M. marinum* as a diagnostic consideration, as geographic and demographic assumptions may contribute to systematic underreporting and prolonged morbidity in at-risk populations, including those with immunosuppression or recent bariatric surgery.

## CRediT authorship contribution statement

**Briccio Cadiz:** Writing – review & editing, Visualization, Supervision, Methodology, Investigation, Formal analysis, Data curation, Conceptualization. **Iyer Karthik:** Writing – review & editing, Visualization, Supervision, Project administration, Methodology, Formal analysis, Conceptualization. **Vikram Oke:** Writing – review & editing, Visualization, Formal analysis. **Bacon Nickolas:** Writing – review & editing, Visualization, Supervision, Methodology, Investigation. **Zackary Houghton:** Writing – review & editing, Writing – original draft, Methodology, Investigation, Formal analysis, Data curation, Conceptualization. **Katherine Raymer:** Writing – review & editing, Writing – original draft, Methodology, Investigation, Formal analysis, Data curation, Conceptualization. **Torrento Marlon:** Writing – review & editing, Supervision, Methodology, Investigation, Formal analysis, Data curation. **Riley Bricker:** Writing – review & editing, Writing – original draft, Methodology, Investigation, Formal analysis, Data curation, Conceptualization.

## Informed consent

Written and informed consent was obtained from the patient for publication of this case report and accompanying clinical photographs.

## Ethical Approval

Ethical approval was not required for this case report in accordance with institutional policy. Written informed consent was obtained from the patient for publication of this case report and any accompanying images.

## Funding

None. This research did not receive any specific grant, contract, or other financial support from funding agencies in the public, commercial, or not-for-profit sectors.

## Declaration of Competing Interest

The authors declare that they have no known competing financial interests or personal relationships that could have appeared to influence the work reported in this paper.
